# Wolff’s law in action: a mechanism for early knee osteoarthritis

**DOI:** 10.1186/s13075-015-0738-7

**Published:** 2015-09-01

**Authors:** Andrew J. Teichtahl, Anita E. Wluka, Pushpika Wijethilake, Yuanyuan Wang, Ali Ghasem-Zadeh, Flavia M. Cicuttini

**Affiliations:** Baker IDI Heart and Diabetes Institute, 99 Commercial Road, Prahan, VIC 3004 Australia; Department of Epidemiology and Preventive Medicine, School of Public Health and Preventive Medicine, Monash University, Alfred Hospital, 99 Commercial Road, Prahran, VIC 3004 Australia; Department of Medicine, Austin Health, University of Melbourne, 145 Studley Roak, Heidelberg, VIC 3084 Australia

## Abstract

There is growing interest in the role of bone in knee osteoarthritis. Bone is a dynamic organ, tightly regulated by a multitude of homeostatic controls, including genetic and environmental factors. One such key environmental regulator of periarticular bone is mechanical stimulation, which, according to Wolff’s law, is a key determinant of bone properties. Wolff’s law theorizes that repetitive loading of bone will cause adaptive responses enabling the bone to better cope with these loads. Despite being an adaptive response of bone, the remodeling process may inadvertently trigger maladaptive responses in other articular structures. Accumulating evidence at the knee suggests that expanding articular bone surface area is driven by mechanical stimulation and is a strong predictor of articular cartilage loss. Similarly, fractal analysis of bone architecture provides further clues that bone adaptation may have untoward consequences for joint health. This review hypothesizes that adaptations of periarticular bone in response to mechanical stimulation cause maladaptive responses in other articular structures that mediate the development of knee osteoarthritis. A potential disease paradigm to account for such a hypothesis is also proposed, and novel therapeutic targets that may have a bone-modifying effect, and therefore potentially a disease-modifying effect, are also explored.

## Background

Historically, osteoarthritis (OA) had been considered a disease predominantly affecting articular cartilage. More recently, it has been appreciated that OA affects the entire joint and all of its constituent tissues and that a spectrum of structural changes ranges from early asymptomatic disease detected on magnetic resonance imaging (MRI) to painful end-stage radiographic disease requiring joint replacement surgery.

There is growing recognition for the role of bone in both early- and late-stage knee OA. Indeed, radiographic criteria for the diagnosis of OA developed in the 1950s included subchondral sclerosis and osteophytes as hallmarks of disease [1]. In the spectrum of knee OA, radiographic changes represent advanced disease, but these evolve over a period of time. Earlier changes in bone are likely to occur before radiographic disease is evident.

Bone is a dynamic tissue that is tightly regulated by a multitude of homeostatic controls. One key environmental regulator of periarticular bone is mechanical stimulation. Wolff’s law relates to the response of bone to mechanical stimulation and states that bony adaptation will occur in response to a repeated load [2]. It is interesting to consider this in the setting of knee OA, which has a strong biomechanical component to its etiology.

When periarticular bone is subjected to increased loading, a number of bone properties change. These include, but are not limited to, an expanding subchondral bone cross-sectional area, changes in bone mass, and remodeling of the trabeculae network. Although these changes likely represent appropriate homeostatic responses of bone to increased loading, they also appear to inadvertently predate maladaptive responses in other articular structures, most notably cartilage. For instance, animal studies have demonstrated that surgical damage to subchondral bone leads to degradation in the overlying articular cartilage in dogs [3] but that increased thickness of the subchondral bone occurs prior to any histologic evidence of cartilage degradation in a guinea pig model of spontaneously occurring knee OA [4].

This review hypothesizes that adaptations of periarticular bone in response to mechanical stimulation cause maladaptive responses in other articular structures that mediate the development of knee OA. Furthermore, this review explores one potential mechanism hypothesizing how bony adaptation to increased mechanical load may inadvertently predate early knee OA. Novel therapeutic agents that may have a bone-modifying effect, and therefore potentially a disease-modifying effect, are also explored.

## Wolff’s law and mechanotransduction

Julius Wolff (1836–1902), a German anatomist and surgeon, theorized that bone will adapt to the repeated loads under which it is placed [2]. He proposed that, if load to a bone increases, remodeling will occur so that the bone is better equipped to resist such loads. Likewise, he hypothesized that, if load to a bone decreases, homeostatic mechanisms will shift toward a catabolic state, and bone will be equipped to withstand only the loads to which it is subjected.

It is now recognised that remodeling of bone in response to a load occurs via sophisticated mechanotransduction mechanisms. These are processes whereby mechanical signals are converted via cellular signaling to biochemical responses [5]. The key steps involved in these processes include mechanocoupling, biochemical coupling, signal transmission, and cell response [6].

## Wolff’s law in action at the knee

Some of the most convincing evidence to substantiate the role of Wolff’s law at the knee has been derived from animal models. Mice with varying loads applied to the tibial diaphysis demonstrated that knee loading was an effective means of enhancing bone formation in a loading frequency-dependent manner [7]. Likewise, a recent mouse study examined an in vivo tibial loading model to determine the adaptive responses of bone to mechanical loading and to assess the influence of load and duration [8]. Cyclic compressive loads were applied to the left tibial knee joint of adult and young mice for 1, 2, and 6 weeks. Mechanical loading promoted increased metaphyseal bone mass in young mice but not in adult mice.

### Subchondral cross-sectional area of bone

#### Biomechanical variables and subchondral bone cross-sectional area

Among humans, some of the earliest work linking knee joint loads and bone adaptation in vivo was born from gait analyses. Gait analyses enable biomechanical variables that estimate knee joint loads to be examined from force platforms. For instance, the external knee adduction moment is a dynamic measure that occurs during the stance phase of human ambulation. It acts to disproportionately distribute joint loads to the medial tibiofemoral compartment [9] and is associated with radiographic knee OA [10]. The first reported association between the knee adduction moment and the cross-sectional area of the tibial plateau was in 2004, when the knee adduction moment during normal walking was significantly correlated with the proximal medial tibial plateau bone cross-sectional area in 20 healthy adult women (r = 0.63, *P* < 0.005) [11]. Since then, the knee adduction moment has consistently been shown to be associated with the medial tibial plateau bone size in knee OA [12, 13]. Mechanistically, this may be an example of Wolff’s law enabling subchondral bone to better dissipate joint loads.

#### Obesity and subchondral bone cross-sectional area

Obesity is another factor that can increase knee joint loading. Indeed, the unequivocal link between obesity and knee OA [14, 15] may be due in part to the added mechanical load imparted to bone by the excessive body mass. In a cross-sectional study with a convenience sample of 372 adults, body mass index (BMI) was positively associated with both the medial (7.1 mm^2^ kg per m^2^, *P* = 0.001) and, to a lesser extent, the lateral (3.2 mm^2^ kg per m^2^, *P* = 0.037) tibial bone area [16]. Those who were obese (BMI ≥30 kg/m^2^) also had greater medial tibial bone area compared with normal weight subjects [16]. This medial compartment predisposition suggests a local mechanical effect and further supports Wolff’s law. Nevertheless, it is also possible that obesity and bone area share common pathways (for example, genetic), such that an increase in one variable (for example, obesity) is paralleled by increases in the other (for example, bone size). However, it is unlikely that a larger tibial bone area is simply reflective of body size, since this has been adjusted for in multivariate analyses.

#### Joint derangement and subchondral bone cross-sectional area

Bone adaptation also occurs when a joint cannot adequately distribute external loads secondary to derangements in articular structures. The primary role of the meniscii at the knee is to cushion and redistribute joint loads. Therefore, when the function of a meniscus is impaired by an anatomical tear or extrusion, or menisectomy, joint load would be expected to increase, with potential ramifications to subchondral bone. It has been shown that meniscal surface area strongly reflects the ipsilateral proximal tibial plateau area [17], and meniscal tears and extrusions are associated with greater bone area [18–20]. Similarly, it has been demonstrated that individuals with anterior cruciate ligament (ACL) tears have significantly larger bone surface areas in the medial tibia and femur [21]. Nevertheless, it is unclear whether increased tibial bone area predisposes structural changes in menisci and ligament or vice versa. This was partly answered in a study of knee OA, wherein baseline medial meniscal extrusion was associated with increased expansion of tibial plateau bone area over the course of 2 years [22].

### Bone mineral density and the trabecular network

Bone mass and density at the knee can be studied by measuring local periarticular bone mineral density (BMD) with dual-energy x-ray absorptiometry and by using fractal analysis to produce a description of trabecular bone microarchitecture based on the cross-linkage, number, and size of trabeculae [23, 24]. The literature examining the response of bone architecture to mechanical loading at the knee is inconsistent. Whereas some studies have shown that periarticular BMD increases with increased mechanical load [25–27], others have demonstrated contrary findings, including a reduction in periarticular BMD [28, 29].

However, the inconsistencies in the response of bone architecture to mechanical load are likely attributable to whether or not significant time has elapsed to enable bony remodeling. For instance, in animal models of induced knee OA, the acute and rapid resorption of periarticular bone [28, 29] may simply be a reflection of the unaccustomed and excessive loads that initially overburden bone. However, after this acute insult, dynamic compensatory changes that increase periarticular BMD likely occur [29]. The early resorptive phase may cause reduced bone mass, predisposing microfractures with subsequent osteoblastic activity and bone healing ultimately increasing BMD. This time-dependent response of bone may explain why, whereas animal models of acute OA demonstrate a reduction in periarticular BMD, epidemiological studies have demonstrated that load is ultimately associated with an increase in periarticular BMD.

#### Periarticular bone mineral density

##### Biomechanical variables and periarticular bone mineral density

Knee alignment is an important determinant of periarticular BMD. Whereas varus deformity is associated with greater medial rather than lateral tibial BMD, valgus deformity is associated with greater lateral tibial BMD [25, 26]. In 69 people with medial tibiofemoral knee OA, the proximal tibial BMD was also correlated with the peak knee adduction moment [26].

##### Obesity and periarticular bone mineral density

There is a paucity of animal and human studies examining the association between obesity and periarticular BMD at the knee. Indeed, few data are available examining the association between obesity and BMD at varied anatomical sites, and despite the popular belief that obesity may be protective against osteoporosis, this is contentious [30–32].

##### Joint derangement and periarticular bone mineral density

Akin to the increases in articular bone area expansion that occurs when menisci are damaged, it has been shown that meniscal damage is also associated with higher regional periarticular tibial BMD in the same compartment [27]. However, in the first 12 weeks after ACL disruption in dogs, there is a rapid decrease in BMD in the periarticular cancellous bone of the knee [28]. This corroborates earlier work demonstrating a marked increase in bone uptake at the knee approximately 5 weeks after ACL disruption in a canine model of knee OA, signifying the marked changes occurring in subchondral bone after joint derangement [33].

#### The trabecular network

##### Biomechanical variables and the trabecular network

Despite the proposition that, in skeletal biology, trabeculae align with the orientation of dominant compressive loads, this has not been widely tested and evidence supporting this hypothesis is derived from animal studies. A study of birds has shown that fine trabecular bone in the distal femur has a high degree of correspondence between changes in joint angle and trabecular orientation, supporting the prediction that trabecular bone adapts dynamically to the orientation of the peak compressive force [34]. Likewise, in mice, load was shown to significantly increase trabecular bone volume at 8 weeks of life, but modified trabecular organization with decreases in trabecular bone volume was demonstrable in 12- and 20-week-old mice [35]. Whether this translates to human bone biology is speculative, given the different kinematics and kinetics of human locomotion.

##### Obesity and the trabecular network

Few studies have examined the association between obesity and trabecular structure at the knee (distal femur and proximal tibia) in either humans or animals. In a study of adolescent black females, it was found that obese people had a lower trabecular compartment area at the distal tibia than their non-obese counterparts [36]. In a study comparing leptin-deficient (ob/ob) and leptin receptor-deficient (db/db) female mice with wild-type mice, it was found that extreme obesity caused by leptin impairment was associated with reduced subchondral bone thickness and increased relative trabecular bone volume in the tibial epiphysis [37]. Nevertheless, reduced activity and joint loading observed among obese subjects may be an alternate explanation (that is, a confounder) to the relationships observed between obesity and trabecular network abnormalities.

##### Joint derangement and the trabecular network

Trabeculae are also affected by joint damage, and it has been shown that there is lower apparent trabecular number with greater apparent residual trabecular thickness and separation in people with meniscal tears [38]. In a model of post-traumatic mouse OA induced by ACL rupture, rapid loss of trabecular bone in the injured knee occurred, followed by a partial recovery of trabecular bone to a new steady state by 28 days after injury [29].

## Evidence for bone adaptation to predate maladaptive changes in articular cartilage

Bone and cartilage are intimately related since the avascular hyaline cartilage is reliant in part on the vascularized subchondral bone to remain viable. Technetium scintigraphy work has demonstrated that increased bone metabolism is related to OA severity [39]. However, this does not address the primary inciting event: does cartilage destruction initiate increased bone metabolism, or vice versa? When animal models of induced OA and natural history studies of humans have used MRI over the past decade [3, 4, 20, 40, 41], they have generally demonstrated that changes in subchondral bone precede cartilage degeneration. For instance, among animal studies, surgical damage to subchondral bone leads to degradation in the overlying cartilage in canines [3]. In another study [4], increased thickness of the subchondral bone occurred prior to any histologic evidence of cartilage degradation in a model of spontaneously occurring knee OA.

### Bone geometry and cartilage

Several in vivo human studies have examined the association between tibial plateau size and changes in cartilage volume. In a population study of 324 adults, larger baseline proximal tibial bone area was associated with increased cartilage volume loss over the course of 2 years at both the medial and lateral tibial sites [40]. Nevertheless, adjusting for baseline cartilage volume or the presence of osteophytes attenuated results, suggesting that the presence of OA or very early structural damage within the joint may be a key mediator of the response of cartilage to a larger bone size. However, it is important to note that these findings were unlikely to be confounded by the stature of a person, since multivariate analyses adjusted for both gender and BMI. In the same population, larger baseline proximal tibial bone area was associated with worsening cartilage defect scores [41]. In symptomatic knee OA, knee cartilage defects tended to progress with time, and increased proximal tibial bone area was a determinant of such progression [20]. Since cartilage defects and volume loss predict the development of radiographic knee OA and joint replacement surgery [42], these data may implicate the tibial plateau size as an important early target in the prevention of knee OA [42].

### Bone mineral density, architecture, and cartilage

Several studies have shown a positive association between BMD at a number of sites, including total body, femur, and spine, and knee cartilage volume [43–45]. However, these studies have not focused on localized periarticular BMD at the subchondral interface of the knee, arguably the most important anatomical region in OA.

In people with established knee OA, it has been shown that the medial-to-lateral tibial BMD ratio was positively associated with medial joint space narrowing [46]. Likewise, periarticular BMD at the knee in people with knee OA was significantly correlated with future joint space narrowing [47] and predicted medial knee cartilage defect development [48]. In an animal model of induced knee OA, mice that had their ACL ruptured demonstrated rapid loss of trabecular bone in the first week after injury but recovered by 28 days [29]. By day 56, there was considerable non-native bone formation and evidence of deterioration in histologic grading of articular cartilage [29].

The trabecular structure of bone also appears to be a determinant of cartilage changes at the knee. In an induced model of knee OA in guinea pigs, trabeculae were thicker and further apart and topographically mirrored untoward histomorphometric changes in cartilage [49]. In women with established knee OA, vertical oriented baseline subchondral trabecular bone integrity of the medial tibia, as determined by fractal analysis, predicted both medial joint space narrowing on radiographs and medial tibial cartilage volume loss from MRI [50]. Likewise, in people with end-stage knee OA requiring total knee replacement surgery, reduced trabecular spacing was correlated with a decreased joint space width [51].

Cartilage response to systemic bone mineral density may vary according to disease status. There is conjecture as to whether a higher systemic BMD is to the benefit or detriment of cartilage. This may differ on the disease state (that is, initiation versus progression of disease). A large radiographic study (*n* = 1,754) demonstrated that high systemic BMD increased the risk for incident knee OA, as measured by the onset of joint space narrowing [52]. However, in the only longitudinal MRI study, a high total body BMD was associated with an increase in femoral cartilage thickness, and a high spine BMD was associated with increases in femoral and lateral tibial cartilage thickness in subjects with OA [53]. This raises the possibility that the influence of bone health parameters, such as BMD, may differ on the disease state whereby a high systemic BMD may predate disease initiation but may help to protect against disease progression.

## A disease paradigm for primary and secondary knee osteoarthritis

**Fig. 1:**
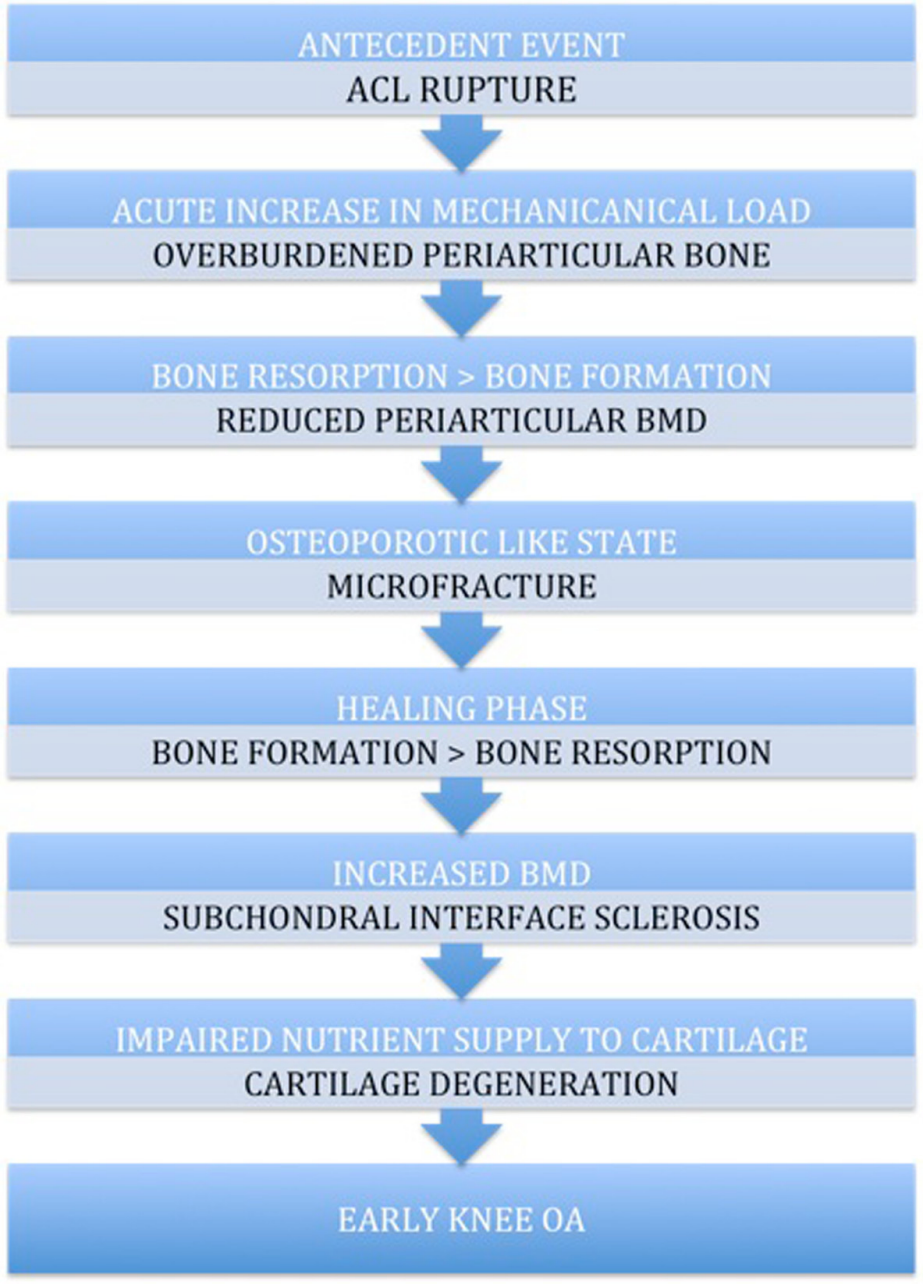
Hypothesized disease paradigm for early traumatic secondary knee osteoarthritis (OA). *ACL* anterior cruciate ligament, *BMD* bone mineral density

Although OA is a heterogeneous disease with multiple pathways of varied etiologies leading to a common endpoint of joint damage, we propose a “bone adaptation” paradigm with a common final pathway of cartilage damage in both primary and secondary knee OA. In secondary knee OA, we base our disease paradigm on evidence collated from animal studies which, via trauma, induce OA (Fig. 1). An initial acute insult to the knee, such as an ACL rupture, leads to a sudden overburdening of periarticular bone and a resorptive phase [28, 29]. This may initially cause a transient “osteoporosis-like” state, as has been demonstrated in dog and mouse models of OA [28, 29]. This overburdening of periarticular osteoporotic bone may result in microfractures and a compensatory shift toward an osteoblastic state to promote fracture healing that ultimately results in an increase in periarticular BMD [28, 29]. These changes therefore appear time-dependent. This is exemplified in a mouse model of OA, in which there was rapid loss of trabecular bone 7 days after knee injury. However, partial recovery of bone to a new state occurred by 28 days, and by day 56, considerable non-native bone formation had occurred [29]. Moreover, in a canine model, the regions of pronounced BMD loss corresponded to the area of focal cartilage defects [28]. In parallel with these changes, trabeculae network remodeling occurs with subsequent downstream effects on cartilage loss [49–51]. Therefore, although these adaptations may ultimately enable bone to better redistribute mechanical load, they inadvertently predispose cartilage degeneration. This may occur in part via impairment of flow of nutrients to the avascular hyaline cartilage via the sclerosed subchondral interface [54], thus setting up a trajectory of early knee OA.

Traumatic secondary OA has an evidence base derived from animal models, but there is a paucity of animal studies examining the more chronic course of primary knee OA. As such, there is less evidence to substantiate any theories underpinning bone changes in primary OA. Nevertheless, we contend that, in primary OA, the disease pattern may follow a more insidious progression that is similar to that hypothesized for secondary OA. In secondary OA, an inciting event such as an ACL rupture causes an acute increase in mechanical load, but we speculate that a more subtle and chronic increase in joint load, such as that imparted by obesity or a change toward knee malalignment, occurs in primary knee OA.

Nevertheless, it is also possible that, in primary OA, a multitude of other bone adaptations which do not invoke a resorptive state are occurring. For instance, rather than resulting in insufficiency fractures, insidious metaphyseal bone expansion of the proximal medial tibial plateau in response to increased joint loads or obesity [11–13, 16] may cause increased subchondral bone area in an attempt to better redistribute joint loads. It is also possible that, as bone area expands, cartilage begins to thin and that fissuring and defects precede volume loss. Alternatively, metaphyseal surface area expansion may impair the mechanical stimulation required for cartilage to remain viable. For instance, in its most extreme form, marked cartilage volume loss occurs after forced immobility such as that induced by spinal cord injury [55, 56]. Therefore, changes in the bone cross-sectional area may enable bone to better redistribute joint loads but may decrease contact pressure on articular cartilage, and insidious but accelerated volume loss may result [56]. Although these paradigms are likely to be oversimplified, the principles of primary and secondary OA may follow a similar trajectory that ultimately ends in an adaptive response of bone but in a maladaptive downstream effect and final common pathway of cartilage loss.

## Potential disease-modifying osteoarthritis drugs

Because periarticular bone changes predate deleterious cartilage changes [20, 40, 41, 57], it has been speculated that drugs targeting bone may have a disease-modifying effect in OA. Indeed, to date, the drugs that arguably show most promise as disease-modifying osteoarthritis drugs (DMOADs) all target bone.

### Anti-resorptive drugs

#### Bisphosphonates

Human studies of progressive knee OA, as determined by joint space narrowing of at least 0.6 mm, demonstrated maintained or improved bony trabecular structure at the knee if risendronate 15 mg/day or 50 mg/week was given, respectively [58]. To the best of our knowledge, three randomized controlled trials have examined the role of bisphosphonates in people with knee OA [59–61]. In the Knee OA Structural Arthritis (KOSTAR) study, a dose-dependent reduction in the level of C-terminal cross-linking telopeptide of type II collagen (CTX-II) was seen in subjects receiving risedronate [60]. Nevertheless, compared with placebo, risedronate did not improve signs or symptoms of knee OA nor did it alter the radiographic progression of disease. The British Study of Risedronate in Structure and Symptoms of Knee OA (BRISK) examined 5 or 15 mg of risedronate in people with mild to moderate medial knee OA over the course of 12 months [61]. The experimental arm in the BRISK study, in contrast to the KOSTAR study, showed significantly greater improvements in pain with a trend toward attenuation of joint space narrowing. Both KOSTAR and BRISK demonstrated reduced CTX-II in the bisphosphonate group, and the BRISK trial also showed reduced urinary N-terminal cross-linking telopeptide of type I collagen, a marker of bone resorption. However, radiographic assessment of the joint space is an insensitive and non-direct measure of articular cartilage. No MRI study has examined cartilage volume as an endpoint in bisphosphonate studies of knee OA. In the only randomized trial which used MRI to investigate subjects with symptomatic knee OA and known bone marrow lesions (BMLs), zolendronic acid was shown to reduce BML size and knee pain at 6 months, and there was a trend toward reduced BML size at 1 year [59].

#### Strontium ranelate

Recent interest in strontium ranelate as a DMOAD stems from a double-blind randomized placebo-controlled trial [62]. In this study, fewer people treated with strontium ranelate demonstrated radiographic progression of knee OA than those treated with placebo, as determined by joint space narrowing. In the Strontium Ranelate Efficacy in Knee Osteoarthritis Trial (SEKOIA), MRI was used to assess patients with knee OA treated with strontium ranelate over the course of 36 months [63]. Compared with placebo, patients who received 2 g/day of strontium ranelate were found to have significantly reduced cartilage volume loss and progression of medial compartment BMLs. In a recent study of dogs with ACL transection, strontium ranelate significantly reduced cartilage lesions at all doses tested (25, 50, or 75 mg/kg per day), but better preservation of the collagen network was seen in dogs that received 50 and 75 mg/kg per day [64]. Subchondral bone thickening observed in placebo dogs was also reduced by strontium ralenate at 50 mg/kg per day.

### Calcitonin

Calcitonin is produced primarily by the thyroid and acts to reduce blood calcium levels, opposing the effects of parathyroid hormone (PTH). Both animal and human studies have shown promising data supporting calcitonin in mitigating the symptoms and structural changes of OA. A recent animal study examined the efficacy of calcitonin on the progression of early-stage OA in a surgically induced model of OA among rabbits [65]. Intramuscular administration of calcitonin delayed the progression of early-stage, surgically induced OA. Similarly, it was shown that transgenic mice overexpressing calcitonin had higher bone volume and were protected against cartilage erosions [66]. Another study examining dogs concluded that by counteracting bone loss, calcitonin reduced cartilage lesions [67].

Among human studies, post-menopausal women with moderate to severe painful radiographic knee OA treated with intranasal calcitonin demonstrated improved pain, stiffness, and function after 3 months of treatment and these improvements remained consistent for 1 year [68]. Moreover, need for rescue analgesia was reduced by approximately 60 % by 12 months of treatment. In a small randomized controlled trial, it was shown that oral calcitonin at a dose of 1 mg/day reduced functional disability and reduced levels of biomarkers CTX-II and matrix metalloproteinase 1 [69].

### Parathyroid hormone

PTH has been shown to act via the type 1 PTH receptor to induce matrix synthesis and suppress maturation of chondrocytes [70]. It was found that, when the ability of PTH analogues to inhibit the terminal differentiation of human articular cartilage in vivo was exploited, a PTH analogue (PTH[1–34]) reduced OA-like changes by decreasing type II collagen and glycosaminoglycan content while increasing type X collagen and chondrocyte apoptosis in rats [70]. Moreover, in a mouse study, PTH[1–34] inhibited aberrant chondrocyte maturation and associated articular cartilage degeneration after induced injury [71]. Whereas immediate systemic administration of PTH[1–34] increased proteoglycan content and inhibited articular cartilage degeneration, delayed treatment induced a regenerative effect. It was concluded that PTH analogues may be useful for decelerating cartilage degeneration and inducing matrix regeneration in people with OA. In a rabbit model of OA preceded by osteoporosis, PTH[1–34] reversed areas of decreased bone area, trabecular thickness, and cartilage damage severity [72]. Human studies are lacking.

## Conclusions

As theorized by Wolff’s law, when knee bone is subject to increasing loads, it responds with compensatory geometrical changes, most notably an expansion in subchondral bone cross-sectional area. Paralleling these geometric changes is the remodeling of bone architecture with a long-term increase in periarticular BMD and remodeling of the trabeculae network. Although these adaptive bone changes help bone to better cope with increased joint loads, they also appear to inadvertently predate maladaptive responses in other joint structures, most notably hyaline articular cartilage.

A potential mechanism for DMOADs may be at the level of various methods of inhibiting bone resorption. After acute injury, such as ACL rupture, animal models of OA have consistently demonstrated a rapid increase in localized periarticular bone resorption. We have speculated that this acute period likely represents an overburdening of bone by a sudden increase in mechanical load. However, as bone adapts, compensatory mechanisms, such as an increase in periarticular BMD, secondary to microfractures may enable the bone to withstand increased bony loads. It is speculated that anti-resoprtive therapies such as bisphosphonates and strontium ranelate may have a DMOAD effect by inhibiting this initial resorptive phase of overburdened periarticular bone. Alternatively, other bone-modifying agents such as calcitonin and PTH analogues are attracting increasing attention as potential DMOADs. Further work is required to examine novel therapeutic agents that may have a bone-modifying effect, and therefore a disease-modifying effect, in knee OA.

## Abbreviations

ACL: Anterior cruciate ligament
BMD: Bone mineral density
BMI: Body mass index
BML: Bone marrow lesion
BRISK: British Study of Risedronate in Structure and Symptoms of Knee OA
CTX-II: Type II collagen
DMOAD: Disease-modifying osteoarthritis drug
KOSTAR: Knee OA Structural Arthritis
MRI: Magnetic resonance imaging
OA: Osteoarthritis
PTH: Parathyroid hormone
